# The nuclear receptor LXR modulates interleukin-18 levels in macrophages through multiple mechanisms

**DOI:** 10.1038/srep25481

**Published:** 2016-05-06

**Authors:** Benoit Pourcet, Matthew C. Gage, Theresa E. León, Kirsty E. Waddington, Oscar M. Pello, Knut R. Steffensen, Antonio Castrillo, Annabel F. Valledor, Inés Pineda-Torra

**Affiliations:** 1 Centre for Clinical Pharmacology, Division of Medicine, University College of London, 5 University Street, London, WC1 E6JF, United Kingdom; 2 Division of Clinical Chemistry, Department of Laboratory Medicine, Karolinska Institut, Huddinge, Sweden; 3 Instituto de Investigaciones Biomedicas “Alberto Sols” Consejo Superior de Investigaciones Científicas (CSIC) de Madrid, Unidad de Biomedicina (Unidad Asociada al CSIC), Instituto Universitario de Investigaciones Biomedicas y Sanitarias (IUIBS) de la Universidad de Las Palmas de Gran Canaria, Las Palmas, Spain; 4 School of Biology, University of Barcelona, Diagonal 643, Planta 3, 08028 Barcelona, Spain

## Abstract

IL-18 is a member of the IL-1 family involved in innate immunity and inflammation. Deregulated levels of IL-18 are involved in the pathogenesis of multiple disorders including inflammatory and metabolic diseases, yet relatively little is known regarding its regulation. Liver X receptors or LXRs are key modulators of macrophage cholesterol homeostasis and immune responses. Here we show that LXR ligands negatively regulate LPS-induced mRNA and protein expression of IL-18 in bone marrow-derived macrophages. Consistent with this being an LXR-mediated process, inhibition is abolished in the presence of a specific LXR antagonist and in LXR-deficient macrophages. Additionally, IL-18 processing of its precursor inactive form to its bioactive state is inhibited by LXR through negative regulation of both pro-caspase 1 expression and activation. Finally, LXR ligands further modulate IL-18 levels by inducing the expression of IL-18BP, a potent endogenous inhibitor of IL-18. This regulation occurs *via* the transcription factor IRF8, thus identifying IL-18BP as a novel LXR and IRF8 target gene. In conclusion, LXR activation inhibits IL-18 production through regulation of its transcription and maturation into an active pro-inflammatory cytokine. This novel regulation of IL-18 by LXR could be applied to modulate the severity of IL-18 driven metabolic and inflammatory disorders.

Interleukin (IL)-18 and IL-1β are highly potent inflammatory cytokines that belong to the Interleukin-1 family of immunomodulators, and are implicated in a range of severe and diverse autoimmune and inflammatory diseases as well as metabolic and vascular disorders including rheumatoid arthritis, diabetes atherosclerosis^1^. IL-18 is primarily secreted by macrophages and dendritic cells^2^ and is constitutively expressed even in the absence of pro-inflammatory stimulation while IL-1β is absent from mononuclear cells and hematopoietic cells from healthy subjects^3^. One of the most prominent IL-18 actions is the induction of interferon gamma (IFNγ ) production by Th1 cells in the presence of IL-12 or IL-15 and thus is considered a pro-inflammatory cytokine. IL-18 also increases Fas Ligand (FasL) on natural killer (NK) cells thereby promoting FasL-mediated cytotoxicity.

IL-18 activity can be regulated at the (1) promoter/transcriptional level, (2) through post-translational processing (inflammasome-dependent cleavage) and (3) indirectly, through the sequestering role of the endogenous inhibitor IL-18 binding protein (IL18-BP)^1^. Despite its documented role on several pathologies, transcriptional regulation of the IL-18 gene remains largely unexplored. It has been previously shown that AP-1, PU.1, Interferon Regulatory Factor 8 (IRF8) as well as nuclear factor (NF)-κ B transcription factors play a critical role in the activation of the IL-18 promoter by LPS in macrophages^4^,^5^,^6^,^7^. Like IL-1β , IL-18 is produced as an inactive precursor (pro-IL-18) that is cleaved into a biologically active cytokine by the inflammasome protease component caspase 1^8^,^9^. Inflammasomes are cytosolic multimeric complexes that are key to innate immune responses and consist of a sensor protein, an adaptor protein and the enzymatic component pro-caspase 1, which itself is subject to proteolytic activation. Inactive pro-caspase 1 is processed by the nucleotide-binding domain and leucine-rich repeat pyrin containing protein-3 (NLRP3) and apoptosis-associated speck-like protein (ASC) inflammasome complex formed upon LPS and adenosine-triphosphate (ATP) activation^10^,^11^. NLRP3 is triggered by many of the metabolic by-products generated in chronic metabolic diseases, including beta-amyloid plaques in Alzheimer’s disease, islet amyloid polypeptide involved in type 2 diabetes and cholesterol, hydroxyapatite and urea crystals involved in atherosclerosis, rheumatoid arthritis and gout, respectively^12^. The negative regulation of caspase 1 is thought to be crucial to a balanced inflammatory response, however this area of research is limited to a mere handful of studies^13^,^14^,^15^,^16^. A potent endogenous inhibitor of IL-18, IL-18 binding protein (IL-18BP) is also secreted to the ECM and directly interacts with IL-18 to inhibit its activity^1^. Most diseases implicating IL-18 actually result from an imbalance between IL-18 and IL-18BP circulating levels^1^. Thus, a finely tuned control of IL-18 and IL-18BP levels by a number of coordinated regulatory mechanisms is required. However, little is known on the gene regulation of IL-18BP levels^17^,^18^.

The nuclear receptors Liver X Receptors (LXRs) are lipid-activated transcription factors expressed in numerous immune cell types including macrophages whose activation modulates immune responses^19^. LXRs modulate gene transcription by heterodimerising with the Retinoid X Receptor (RXR) and binding to specific DNA sequences termed LXR response elements (LXREs) in the transcriptional regulatory regions of their target genes^19^. LXR transcriptional activity is induced by certain oxysterols and synthetic compounds including T0901317^20^ or GW3965^21^. Both LXR isoforms, LXRα and LXRβ , control macrophage cholesterol homeostasis and regulate macrophage inflammatory responses, phagocytosis and apoptosis^22^,^23^. LXR activation prevents macrophage cholesterol accumulation by inducing ABC transporter-mediated cholesterol efflux through the transcriptional regulation of ABCA1 and ABCG1 and limits cholesterol uptake. Furthermore, LXRs inhibit the transcription of pro-inflammatory genes (including IL-6, IL-1β and iNOS) lacking LXREs in their promoters or enhancers. They do so by antagonising the activity of transcription factors such as NF-κ B or AP1 without directly binding to DNA themselves, in a process termed transrepression^23^. IL-1β can be regulated by LXRs through this mechanism^24^ and in this way LXR activation inhibits inflammatory responses in macrophages and other immune cells including lymphocytes^25^. The postulated molecular mechanism underlying this regulation involves the SUMOylation of the receptor upon ligand binding, inhibiting LPS-induced corepressor clearance from the promoter of inflammatory genes^26^ although this mechanism has been recently challenged^27^. In addition to IL-1β , LXRs regulate multiple inflammatory, metabolic and vascular conditions where dysregulated IL-18 levels are observed^1^,^25^. However, whether LXR activation modulates IL-18 levels remains unknown.

In this study we demonstrate the novel negative regulation of IL-18 levels by LXR at multiple regulatory checkpoints: its expression, activation and bioavailability. We show for the first time that LXRs inhibit IL-18 gene expression and negatively regulate caspase 1 levels (and hence IL-18 processing and activation by the inflammasome). Moreover, through regulation of IRF8, LXR activation enhances the expression of IL-18BP thus also identifying IL-18BP as a novel IRF8 and LXR target gene. Collectively, LXRs exert multiple actions on IL-18 and IL-1β regulation.

## Results

### IL-18BP is a novel LXR target gene

**Figure 1:**
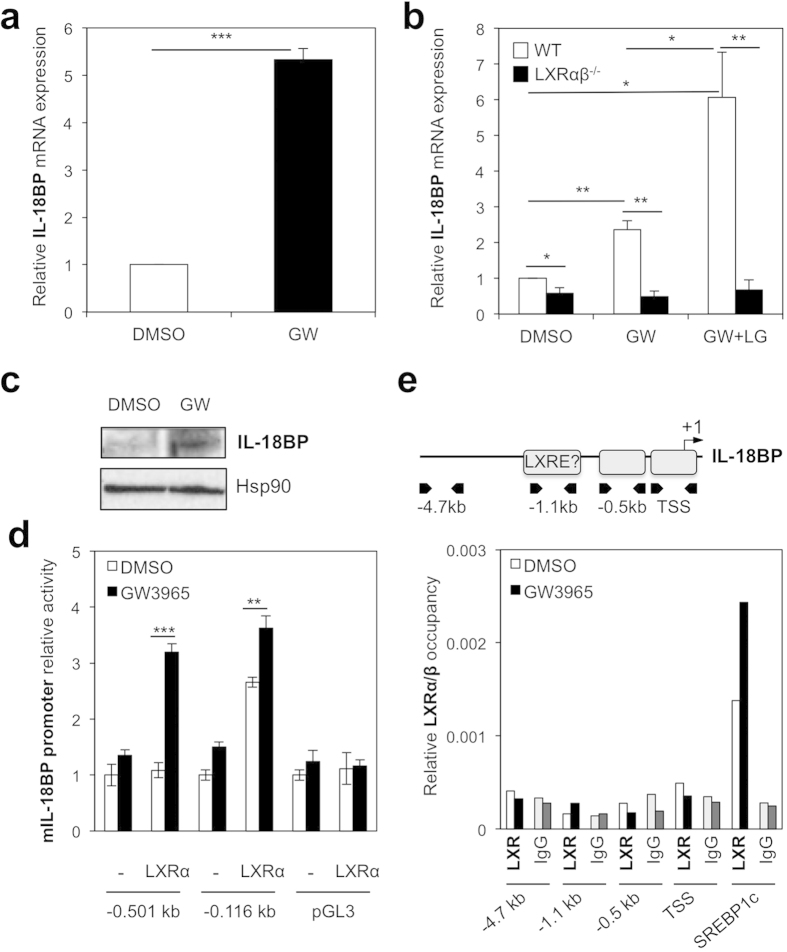
Identification of IL-18BP as a novel LXR target gene. (**a**) BMDM were treated with vehicle (DMSO) or GW3965 (GW) for 24 hours and IL-18BP mRNA content was analyzed by RT-qPCR. Values indicate expression normalized to cyclophilin and are presented relative to the expression in vehicle-treated cells. Values represent the mean of 4 independent experiments ±  SEM. *t*-test: ***p ≤  0.001. (**b**) BMDM from WT or LXRα β deficient mice (LXRα β ^−/−^) were treated as in A with or without the RXR activator LG268 (LG). IL-18BP mRNA content was analyzed as in A. Values represent the mean of 3 independent experiments ±  SEM. *p ≤  0.05, **p ≤  0.01. (**c**) BMDM were treated with vehicle (DMSO) or GW3965 (GW) for 24 hours supplemented with Brefeldin A for the last 6 hours. Protein content was analyzed by immunoblotting and Hsp90 levels were assayed as loading control. (**d**) RAW264.7 cells were transfected with hLXRα or pcDNA3.1 (− ) along with the indicated IL-18BP luciferase reporters or pGL3 empty vector. Cells were treated as described in the Methods section. For each reporter, luciferase and β -galactosidase activities were measured and the ratio was compared to the vehicle-treated condition in the absence of LXRα (− ), which was set as 1. Data are mean value ±  SD (n =  3) of one representative experiment. *t*-test: **p ≤  0.01, ***p ≤  0.001. (**e**) Upper panel, location of a putative LXRE in the IL-18BP locus. Arrows indicate the position of primers used for ChIP assays. Lower panel, BMDM cells were incubated with or without GW3965 at 1 μ mol/L for 2 hours. LXR occupancy was assessed by ChIP assays. Primers shown in upper panel were used to amplify an unrelated site at − 4.7 kb that served as a negative control, the − 1.1 kb putative LXRE, − 0.5 kb and TSS as GW3965 responsive sites. SREBP1c primers were used as a positive control for LXR binding. Shown is a representative experiment of three.

IL-18 binding protein (IL-18BP) is a potent IL-18 endogenous inhibitor secreted by macrophages that directly neutralises IL-18 with a high level of affinity (Kd =  400 pM) at a 1:1 stœchiometry^1^. We assessed the effect of ligand-activated LXRs on IL-18BP gene expression in BMDM. BMDM offer advantage over other murine *in vitro* macrophage models as activation experiments can be performed on a homogenous population of quiescent cells^28^. Treatment with GW3965 ligand increased IL-18BP mRNA expression by almost 6-fold compared to control mouse macrophages (Fig. 1a). This effect was further enhanced by the RXR agonist LG268 in wild type (WT), but not in LXR-deficient, macrophages (Fig. 1b). Consistently, LXR ligand enhanced secretion of IL-18BP by macrophages (data not shown) and increased intracellular IL-18BP protein levels in these cells in the presence of Brefeldin A, an inhibitor of Golgi apparatus secretion (Fig. 1c). Thus, LXR activation enhances IL-18BP expression at the mRNA and protein level in primary macrophages in an LXR-dependent manner.

Nuclear receptors LXRα and LXRβ are ligand-activated transcription factors that often bind directly to regulatory regions in their target genes to activate their transcription. To further understand the LXR-mediated molecular mechanism underlying the regulation of IL-18BP gene expression, we first assessed the effect of GW3965 on IL-18BP promoter activity. RAW264.7 macrophage-like cells were transfected with an LXRα expression vector together with luciferase reporters driven by various fragments of the IL-18BP promoter and were subsequently activated with the LXR ligand GW3965 (Fig. 1d). IL-18BP promoter activity was specifically enhanced by the LXR ligand primarily in LXRα -overexpressing cells. The activity of the IL-18BP promoter-driven reporter with only 116 bp upstream of the transcription start site (TSS) however, was enhanced by LXRα even in the absence of ligand and was further increased in the presence of GW3965. This suggests the shorter IL-18BP promoter fragment is more sensitive to LXRα overexpression. We then assessed whether LXRs directly bind this LXR and GW3965-responsive region in the IL-18BP promoter by chromatin immunoprecipitation (ChIP) assays, compared to the LXRE described for SREBP1c^29^. A region at − 4.7kb in the IL-18BP gene was used as a negative control. Unexpectedly, LXRs did not bind either the IL-18BP proximal promoter region (TSS and − 0.5kb), or the − 1.1kb region bearing a strong putative LXR Response Element (Fig. 1e). By contrast, LXR occupied the SREBP1c promoter in vehicle- and ligand-activated cells as expected. Altogether, these results demonstrate that LXRα increases IL-18BP expression by activating the proximal IL-18BP promoter without directly binding this region, suggesting an additional transcription factor is involved in the regulation of IL-18BP by LXR.

### LXR-mediated regulation of IL-18BP expression is dependent on IRF8

**Figure 2:**
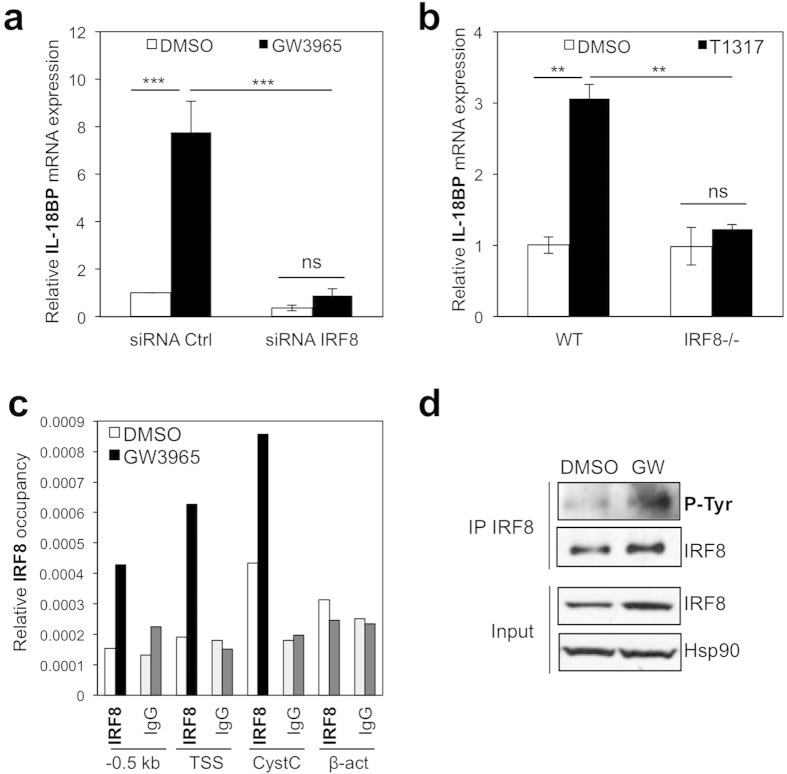
Regulation of IL-18BP expression is dependent on IRF8. (**a**) BMDM were transfected with control (Ctrl) or IRF8 siRNAs for 24 hours and treated vehicle (DMSO) or GW3965 (1 μ mol/L) for 24 hours. IL-18BP mRNA content was analyzed by RT-qPCR. Values indicate expression normalized to cyclophilin and are presented relative to the expression in siRNA-control vehicle-treated cells, which was set as 1. A representative experiment is shown (mean ±  SD) *t*-test: ***p ≤  0.001. (**b**) BMDM from WT or *IRF8*^−/−^ mice were cultured with T1317 (T) (1 μ mol/L) for 24 hours. IL-18BP mRNA content was analyzed by RT-qPCR. Values indicate expression normalized to cyclophilin and are presented relative to the expression in siRNA-control vehicle-treated cells, which is set as 1. A representative experiment is shown (mean ±  SD) *t*-test: **p ≤  0.01. (**c**) BMDM were incubated as in Fig. 1E. IRF8 occupancy was determined by ChIP assays. Primers shown in Fig. 1E were used to amplify the − 0.5 kb and TSS putative IRF8 binding sites. Primers amplifying Cystatin C (CystC) and β -actin (β -act) were used as positive and negative controls, respectively. Background occupancy using IgG in the absence or presence of ligand is depicted by light and dark grey bars respectively. Shown is a representative experiment. (**d**) RAW-LXRα cells were treated with vehicle (DMSO) or GW3965 (GW) (1 μ mol/L) for 24 h. IRF8 was immunoprecipitated and tyrosine phosphorylation of IRF8 was analysed by immunoblotting. Protein content in the cell lysate prior to immunoprecipitation (Input) was analysed. A representative experiment is shown.

We have previously shown that the hematopoietic transcription factor IRF8 mediates the LXR ligand induced expression of arginase 1 in macrophages, in an LXR binding independent manner^30^, a regulation that may contribute to the anti-inflammatory properties of LXRs^31^. LXR ligand induction of IL-18BP mRNA levels is abolished in cells with knocked-down levels of this factor or lacking IRF8, demonstrating this transcription factor mediates LXR activation of IL-18BP expression (Fig. 2a,b). Interestingly, IL-18BP up-regulation by LXR persists even in a pro-inflammatory environment (Fig. S1a). The decrease in IL-18BP upon LPS treatment is associated with reduced levels of IRF8 expression in these cells, thus emphasizing the role of IRF8 on the regulation of IL-18BP (Fig. S1b). However, when macrophages were primed with low levels of IFNγ , expression of IL-18BP and IRF8 was not altered while LXR ligand induced their transcription. These data suggest that different inflammatory pathways affect basal and LXR ligand-activated IL-18BP expression in a different manner, which could have consequences when modulating IL-18 active levels.

*In silico* analysis of the IL-18BP promoter sequence identified two putative Interferon Sensitive Response Elements (ISREs) located at the TSS and − 0.5kb upstream of the TSS. As previously shown, IRF8 binding to Cystatin C, an ISRE-containing gene^32^, was observed in the absence of LXR ligand and was further enhanced upon LXR activation (Fig. 2c), a likely consequence of the increase in IRF8 expression by LXR^30^ (and Fig. S1). While IRF8 was not detected in the IL-18BP promoter at both putative sites tested in vehicle-treated cells, IRF8 occupancy strongly increased upon LXR activation (Fig. 2c). This demonstrates that LXR activation enhances IRF8 binding to the IL-18BP promoter, ultimately regulating IL-18BP levels. We previously showed that LXR directly regulates IRF8 expression and promotes IRF8 heterodimerisation with the transcription factor PU.1^30^. Dimerisation of IRF8/PU.1 and binding to their target genes is enhanced by phosphorylation of IRF8 at Tyr211 in LPS-activated macrophages^2^. Interestingly, IRF8 tyrosine phosphorylation was enhanced by the GW3965 ligand (Fig. 2d), suggesting that regulation of IRF8 tyrosine phosphorylation may also be an important component in the overall regulation of IRF8 activity by LXR.

### LXRs inhibit LPS-induced IL-18 levels

**Figure 3:**
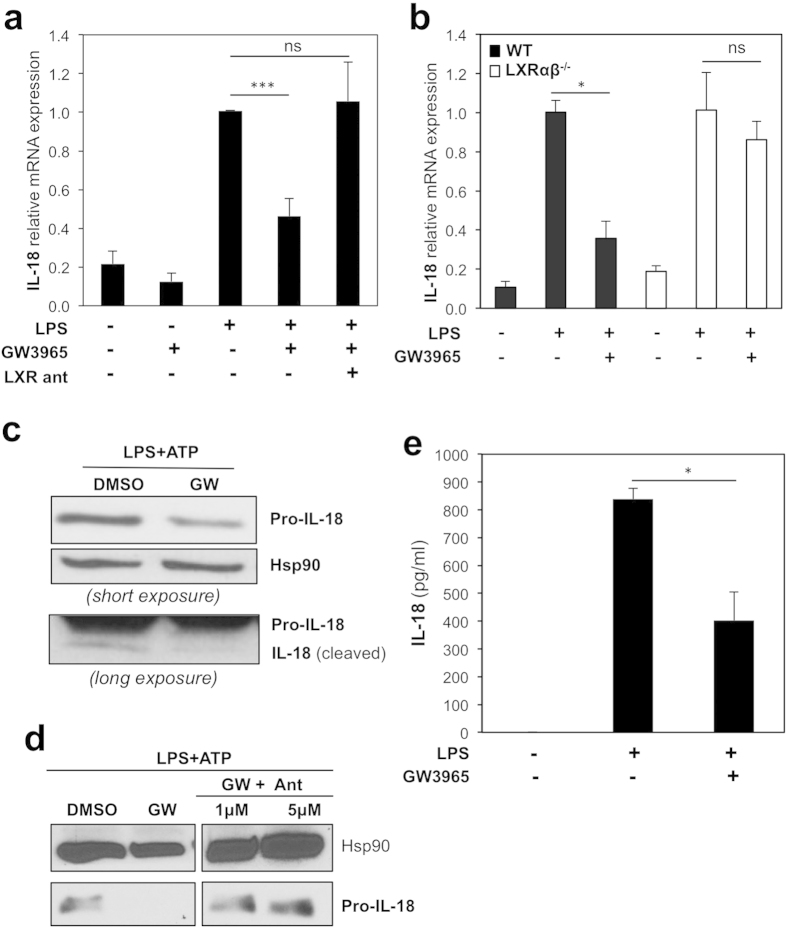
LPS-induced IL-18 levels are regulated by LXR activation. (**a**) BMDM were treated with vehicle (DMSO), GW3965 (1 μ mol/L) or LXR antagonist (LXR ant, 1 μ mol/L) for 24 hours with or without LPS (100 ng/ml) for the last 6 hours. IL-18 mRNA levels were analyzed by RT-qPCR. Values indicate expression normalized to cyclophilin and are presented relative to the expression in LPS-treated cells, set as 1. Values represent the mean ±  SEM (3–4 different BMDM preparations). *t*-test: ***p ≤  0.001, ns *p* >  0.05. (**b**) IL-18 mRNA expression in BMDM from WT and LXRα β ^−/−^ mice were analyzed by RT-qPCR. Shown is a representative experiment performed in triplicate (mean ±  SD) *t*-test: *p ≤  0.05, ns *p* >  0.05. (**c**) BMDM were treated as in A and then activated with ATP for the last 2 hours. Pro-IL-18 expression was analyzed by immunoblotting and Hsp90 levels were assayed as loading control. Shown is a representative experiment of three. (**d**) BMDM were treated as in A. Intracellular expression of IL-18 is shown as analyzed by immunoblotting. Samples shown were analysed on non consecutive lanes of the same blot. (**e**) BMDM were treated as indicated. Secreted IL-18 levels in culture supernatants were quantified by ELISA. Values represent mean ±  SEM (n =  3). *t*-test: *p ≤  0.05.

LPS stimulation enhances the expression of IL-1 family members such as IL-1β and IL-18 through the activation of a PARP1-ERK-NF-κ B-dependent pathway in mouse macrophages^33^. As LXRs antagonise NF-κ B-mediated activation of pro-inflammatory genes in these cells^24^,^26^, we hypothesized these receptors also regulate IL-18 expression. IL-18 mRNA levels increased in the presence of LPS as described^34^, confirming a robust inflammatory response in BMDM. In LPS-activated macrophages, IL-18 mRNA levels were significantly inhibited upon LXR ligand activation by GW3965 compared to cells cultured in vehicle-supplemented medium (Fig. 3a). This was abolished in the presence of the LXR antagonist GSK1440233^35^ or in macrophages from LXRα β ^−/−^ mice (Fig. 3b) demonstrating this inhibition is exerted in an LXR-dependent manner. LXR activation also diminished IL-18 gene expression in IFNγ -stimulated macrophages (Fig. S2a,b), further corroborating the inhibitory effect of LXR on pro-inflammatory cytokine-stimulated IL-18 expression. IL-18 is a secreted cytokine and its maturation and secretion is in part dependent on the activation of the NLRP3 inflammasome. LXR agonists also reduced the intracellular LPS-induced expression of the 24 kDa precursor form (pro-IL-18) and cleaved (active) IL-18 protein expression in the presence of ATP, a known NLRP3 inflammasome activator^36^ (Fig. 3c and S3a). This effect was also dependent on LXR activity as it was abolished in the presence of the LXR antagonist (Fig. 3d). Consistently, LXR ligands significantly decreased LPS and ATP-induced IL-18 levels in macrophage supernatants in a similar manner (Fig. 3e and S4b). Altogether, these results demonstrate that LXR inhibits mRNA and protein expression of pro-IL-18 and secreted levels of bioactive IL-18 in primary macrophages.

### LXRs regulate IL-18 maturation

**Figure 4:**
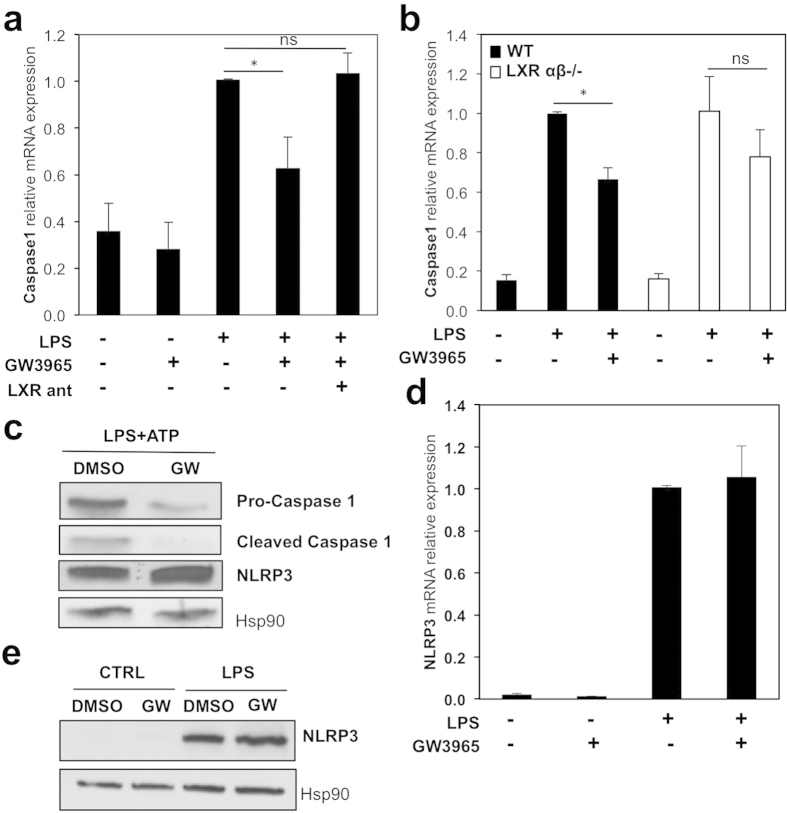
Caspase 1 expression is reduced in response to LXR activation. BMDM were treated with vehicle (DMSO), GW3965, LXR antagonist (LXR ant) or LPS as indicated in Fig. 3A. (**a**) Caspase 1 mRNA expression was analyzed by RT-qPCR. Values indicate expression normalized to cyclophilin and are presented relative to the expression in LPS-treated cells. Values represent the mean ±  SEM (n =  3–4). *t*-test: *p >  0.05, ns *p* >  0.05. (**b**) Caspase 1 mRNA levels in BMDM from WT and LXRα β ^−/−^ mice were analyzed by RT-qPCR. Shown is a representative experiment performed in triplicate (mean ±  SD) *t*-test: *p ≤  0.05, ns *p* >  0.05. (**c**) BMDM were treated with or without GW3965 and LPS for the final 6 hours with addition of ATP for the last 2 hours. Pro-caspase 1, cleaved caspase 1 and NLRP3 protein content were analyzed by immunoblotting and Hsp90 levels were used as loading control. (**d**) NLRP3 mRNA expression in LXR-ligand treated BMDM was analysed as in B. Shown is a representative experiment of 3 (mean ±  SD). (**e**) NLRP3 protein levels were analyzed by immunoblotting. For (**c,e**), representative experiments of 2–3 independent assays are shown.

Processing the inactive pro-IL-18 precursor into a cleaved active IL-18 form in response to pro-inflammatory signals is exerted by a number of processing proteases. One of the best characterized is caspase 1, which cleaves and thus promotes the release of IL-18 from activated macrophages^8^,^9^. We hypothesized that irrespective of the transcriptional repression of the 24 kDa IL-18 precursor, which consistently occurs even when caspase 1 is inhibited (Fig. S4a), LXR activation affects IL-18 maturation by modulating caspase 1. Treatment with the LXR specific agonist GW3965 significantly inhibited caspase 1 gene expression in LPS-activated macrophages in an LXR-dependent manner, as the repressive effect was impaired in the presence of an LXR antagonist or in macrophages lacking LXRα β (Fig. 4a,b). Caspase 1 activation itself is regulated by the canonical inflammasome constituted by NLRP3/ASC^10^,^11^. In response to LXR ligand activation, intracellular expression of both the 45 kDa pro-form and the 20 kDa cleaved form of caspase 1 was reduced in the presence of the inflammasome activator ATP, suggesting that part of the reduction in cleaved IL-18 levels is likely due to diminished levels of caspase 1 (Fig. 4c and S3b,c). Interestingly, the regulation of the pro-caspase 1 form by the GW ligand was blocked by the LXR antagonist (Fig. S4d), demonstrating LXR-dependent effects by GW in these cells. Finally, in contrast to the regulation of IL-18 and caspase 1, NLRP3 expression was unaffected by LXR ligand activation (Fig. 4c–e), indicating the LXR inhibition of IL-18 is independent of a direct effect on the NLRP3 component of the inflammasome. Overall, our results demonstrate that LXR activation decreases IL-18 maturation in part by downregulating caspase 1 expression and activation.

### LXRs inhibit LPS-induced IL-1β levels in bone marrow-derived macrophages

**Figure 5:**
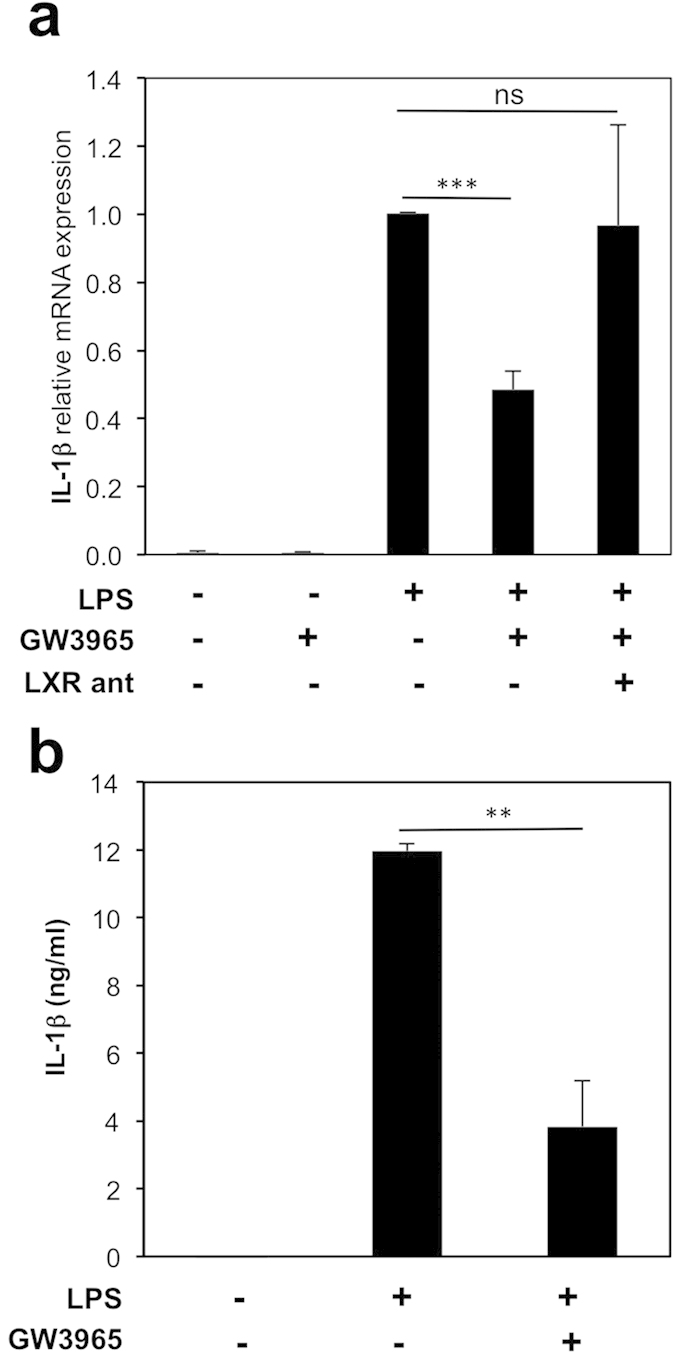
LPS-induced levels of IL-1β are inhibited by LXR activation. (**a**) BMDM were treated with vehicle (DMSO), GW3965 (1 μ mol/L) or LXR antagonist (LXR ant) for 24 hours with or without LPS (100 ng/ml) for the last 6 hours. IL-1β mRNA levels were analyzed by RT-qPCR. Values indicate expression normalized to cyclophilin and are presented relative to expression in LPS-treated cells, set as 1. Values represent the mean ±  SEM (n =  3–4). *t*-test: ***p <  0.001, ns *p* >  0.05. (**b**) Secreted IL-1β levels in cell supernatants were quantified by ELISA. Values represent the mean ±  SEM (n =  3) **p <  0.01.

Another IL-1 family member whose expression is enhanced by LPS stimulation in an NF-κ B-mediated fashion is IL-1β 33. LXR ligands have been shown to antagonize the activation of IL-1β in thioglycolate-ellicited peritoneal macrophages^24^,^26^,^37^. We next examined whether, in addition to IL-18, LXR activation modulates IL-1β expression in BMDMs, which are morphologically and phenotypically different from peritoneal macrophages and present a different cytokine expression profile^38^. LPS-stimulated IL-1β mRNA and secreted protein levels were inhibited upon LXR ligand activation by GW3965 in BMDMs (Fig. 5a,b) demonstrating that LXR regulated pathways involved in the production and maturation of IL-18, are also affecting IL-1β levels. In addition, LXRα β -deficient macrophages secrete enhanced levels of both IL-18 and IL-1β (Fig. S5a,b) further corroborating the role of LXR in the regulation of these inflammasome-activated cytokines.

Overall, this study shows for the first time that LXR activation inhibits IL-18 production in macrophages through regulation of its transcription and processing into an active pro-inflammatory cytokine, as well as by inducing the expression of its potent endogenous inhibitor IL-18BP through the regulation of IRF8. This could have important consequences in the modulation of various inflammatory, metabolic, and vascular disorders by LXR.

## Discussion

IL-18 is a potent pro-inflammatory molecule implicated in a number of inflammatory and autoimmune diseases including rheumatoid arthritis, multiple sclerosis and psoriasis as well as inflammatory and metabolic diseases including atherosclerosis^1^,^39^. IL-18 is a member of the IL-1 family of central mediators of innate immunity and inflammation and thus its tight regulation *via* receptor antagonists, decoy receptors and signaling inhibitors is needed to ensure the right balance between amplification of innate immunity and uncontrolled inflammation^1^. However, there is relatively little insight into its negative regulation. LXRs modulate the severity of conditions where dysregulated IL-18 levels are observed^25^, therefore we hypothesized that LXRs may affect these partly by regulating IL-18. In this report we describe for the first time IL-18 regulation at multiple points by LXRs, including its transcription, activation and bioavailability through regulation of the endogenous IL-18 inhibitor IL-18BP.

Our studies have primarily focused on the regulation of IL-18 in mouse bone marrow derived macrophages, which have been previously used to investigate LXR mode of action^27^,^28^,^29^. The majority of work studying the role of LXRs has been performed in mouse cells and despite some reported species-specific differences, important similarities on LXR actions between human and mouse cells exist^40^. Interestingly, experiments performed in human monocyte-derived macrophages suggest the actions reported here are relevant to a human setting (Fig S6).

LXR agonists robustly inhibited macrophage IL-18 mRNA levels and promoted IL-18BP levels and these effects were shown to be specific for LXR through the use of a combination of LXR-deficient cells and specific antagonists. Furthermore, LXR activation elicited a significant reduction in the LPS-stimulated pro-protein and in the cleaved and secreted bioactive levels of IL-18. In addition, IL-18BP is regulated by the GW ligand in human macrophage cells in an LXR-dependent manner (Fig. S6a) and the expression of the IRF8 mediator of this regulation is also induced in response to LXR ligands as previously shown^30^ (Fig. S6b). In agreement with our findings in mouse macrophages and LXR being a regulator of IL-18 and IL-1β , LPS-activated levels of these cytokines are exacerbated in human macrophages in the absence of the LXR receptors (Fig. S6c,d). This confirms previous studies demonstrating that LXR inhibits the expression of IL-1β as well as other cytokines and chemokines in LPS-stimulated human lung macrophages and the THP1 human monocytic cell line^41^. This work led to the suggestion that LXR ligands could be used for the treatment of lung diseases involving chronic lung inflammation mediated by macrophages. Indeed, LXR activation by GW3965 has shown anti-inflammatory effects (reduction of CXCl10 and CCL5 levels) in lung macrophages from chronic obstructive pulmonary disease patients^42^. In addition to responses to pro-inflammatory stimuli, LXRs have been reported to affect human lymphocyte activation and proliferation, dendritic cell migration, macrophage bacterial and viral infection and their phagocytic capacity to efficiently clear apoptotic cells (reviewed in^40^). Further supporting a role of LXRs in human immunity, a number of studies have linked LXR gene variants with immune disorders including susceptibility to tuberculosis, inflammatory bowel disease, systemic lupus erythematosus and rheumatoid arthritis^40^. However, the functional effects of some of the variants reported remain to be elucidated.

Previous investigations into the negative regulation of IL-18 are limited to observations of indirect mechanisms through caspase 1 modulation^43^ or inhibition by IL-18BP^1^. Our study demonstrates the direct negative regulation of IL-18 gene and protein expression. This work also extends previous findings demonstrating that LXR-mediated regulation of IL-1β is not limited to activated peritoneal macrophages.

Our data further reveal a modulatory role for LXR regulation on the inflammasome through the modulation of caspase 1^8^,^9^. LXR activation significantly and specifically reduced mRNA and protein expression of the precursor form of caspase 1. Inflammasome regulation must be tightly controlled. However, previous investigations on the negative regulation of this complex have mainly focused on inhibitors of the sensor protein e.g. NLRP3, with limited reports on the transcription of the individual components (reviewed in^43^). Studies documenting negative regulation of caspase 1 are also scarce^13^,^14^,^15^,^16^. Recently, LXR was reported to induce caspase 1-dependent cell death in human colon cancer cell lines providing a mechanistic basis underlying some of the anticancer actions of LXR^44^. The mechanism involved however, is fundamentally different from the one we are proposing now for LPS-activated macrophages. According to this study, colon cancer cell death by LXR ligands implicated the non-transcriptional regulation of caspase 1 activation through the direct interaction of LXRβ with the membrane channel pannexin-1, leading to an induced release of ATP and subsequent pyroptosis or caspase 1-induced cell death. Of note, this study involves LXR activation with high concentrations (20 μ mol/L) of the less specific LXR agonist T0901317^20^,^45^. In addition, this membrane channel has been shown to be required for ATP release in cells undergoing apoptosis but not for inflammasome activation *per se*^46^. Other differences between this report and our study are likely due to cell type, cell proliferating status, agonist type and/or the agonist concentration employed. We have used the most specific synthetic LXR agonist, GW3965^21^,^25^, at a lower concentration (1 μ mol/L), in order to avoid off-target effects. It is worth noting that IL-18 processing is not exclusive of caspase 1 since other caspases produce a bioactive IL-18^47^. Following recognition of Gram-negative bacteria, caspase 11 activation (caspase-4 in humans) results in non-canonical inflammasome activation and the subsequent release of IL-18^48^. Additionally, processing of IL-18 can also be exerted upon FasL stimulation in the absence of caspase 1^49^. Whether caspase 11 or FasL are also targeted by LXRs remains to be investigated.

The transcription factors that play a critical role in the activation of the IL-18 by LPS in macrophages include AP-1 and the hematopoietic transcription factors PU.1 and IRF8^4^,^5^. IRF8 is a critical factor in lineage determination and the development of myeloid cells from common progenitor cells^50^,^51^,^52^. Indeed, IRF8 deficiency in mice is characterised by the massive accumulation of immature myeloid cells promoting the development of a chronic myelogenous leukemia-like phenotype^51^ and in humans, loss of function of IRF8 leads to monocytic and dendritic cell immunodeficiency^53^. Additionally, IRF8 is an important mediator of transcriptional responses to LPS and IFNβ 54, reflected by the high susceptibility to intracellular pathogens shown in IRF8-deficient mice^51^.

**Figure 6:**
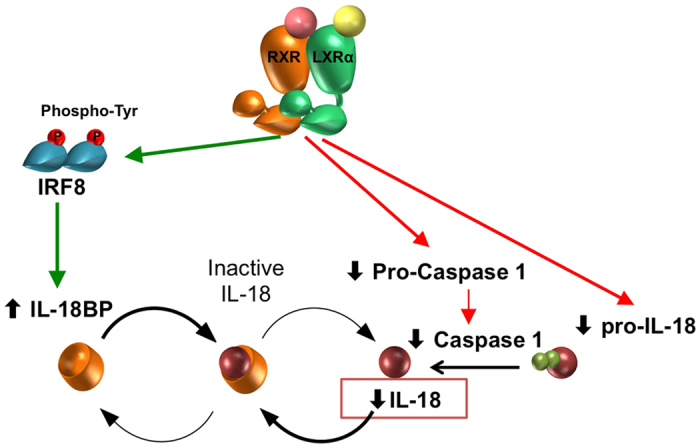
Schematic representation of IL-18 regulation by LXR. Schematic representation of multiple negative regulatory points of LXR activation on IL-18 expression, maturation and bioavailability.

We previously showed that LXRα affects gene expression through IRF8 by directly inducing IRF8 levels and promoting IRF8 dimerization with PU.1^30^, a mechanism that may contribute to the anti-inflammatory properties of LXRs^31^. We now demonstrate that LXR activation enhances IL-18BP expression in an IRF8-dependent manner, thus further implicating this hematopoietic factor as an important modulator of LXR actions beyond its role as a positive mediator of LPS and IFNβ responses. As we showed before for arginase 1^30^, LXR agonists induced the binding of IRF8 to newly identified binding sites on the IL-18BP promoter that are otherwise unoccupied by these factors in untreated conditions. This suggests that LXR activation may expand the IRF8 cistrome, which may have important implications on disease progression when exposing macrophages or possibly other immune cell types to LXR ligands. Indeed, IRF8 was recently shown to bind distinct sets of binding sites, in resting macrophages and in response to LPS stimulation^54^. Whether a similar genome wide activation or redistribution of IRF8 binding sites occurs in response to LXR activation remains to be investigated. Specifically for macrophage IL-18 signalling, a differential activation of binding sites by LXR could explain how the same transcription factor, IRF8, is implicated both in the induction of IL-18 gene expression in response to LPS^4^,^5^ as well as in the expression of its endogenous inhibitor IL-18BP. This IRF8 upregulation of the potent IL-18 endogenous inhibitor elegantly ties in with the LXR reduction of IL-18 expression and activation demonstrated, illustrating an unprecedented negative regulatory role for LXR on this pro-inflammatory cytokine (Fig. 6).

Transcription of inflammasome genes and subsequent IL-18 maturation is primarily regulated by Toll-like receptor (TLR)-dependent pathways^55^. LXRs have been shown to negatively regulate TLR signaling (namely, TLRs 2, 4 and 9) in mouse macrophages^23^ and they likely reduce IL-18 mRNA expression by interfering with these pathways. LXR activation has been shown to dampen LPS -driven inflammation^24^ through a mechanism termed ‘transrepression’ that does not involve the direct binding of LXR to promoter DNA. This involves the SUMOylation of LXR in response to its agonist, which leads to the stabilisation of NCoR transcriptional corepressor complexes on NF-κ B-bound promoters of inflammatory genes, thus actively inhibiting NF-κ B target gene transcription^26^. An alternative mechanism was recently proposed that rather involves changes in cellular lipid metabolism and local membrane composition and organisation^27^. This can occur in the absence of LXR SUMOylation and requires the expression of the cholesterol transporter ABCA1, which is a well characterised positively regulated LXR target gene. It was also shown that ABCA1 represses TLR-induced NFkB and MAPK activation by altering the cholesterol content of membrane domains. Further investigations will be required to identify the precise mechanisms responsible for the repression of the IL-18 and Caspase 1 precursors by LXR described in this study.

In summary, this report offers multiple fundamental new insights into the anti-inflammatory mechanisms of LXRs through the negative regulation of IL-18 at the level of transcription, protein activation and its bioavailability after secretion. This raises the therapeutic potential of LXR to treat the multiple diseases in which dysregulated IL-18 levels contribute to.

## Methods

### Cell culture and animal models

RAW264.7 or RAW-LXRα cells overexpressing LXRα were generated as described in^56^. Bone Marrow-derived Macrophages (BMDM) from C57BL/6 mice were prepared as in^30^ or^57^ using L929 Conditioned Medium (or LCM) as a source of M-CSF for the differentiation of the macrophages. LXRα β ^−/−^ mice^58^ were kindly provided by Dr. David Mangelsdorf (UT Southwestern). Expression of macrophage markers as assessed by flow cytometry analysis was similar in wild-type or LXR-deficient macrophage cultures (data not shown). After 7 days of differentiation, LCM-containing medium was removed, cells were washed with PBS and were then allowed to rest a minimum of 16h in DMEM containing low-endotoxin (≤ 10 EU/mL) FBS and 20 μ g/mL gentamycin without any LCM (activation medium) before being treated with DMSO, 1 μ mol/L T1317 (Sigma) or 1 μ mol/L GW3965 (Tocris) with or without 1–5 μ mol/L LXR antagonist (GSK1440233)^35^. All animal experiments were done in accordance to guidelines and regulations approved by the United Kingdom Home Office. For human monocyte-derived macrophage preparation, human peripheral blood mononuclear cells were isolated from blood of healthy donors by Ficoll density gradient centrifugation. CD14^+^ monocytes were isolated using the EasySep CD14^+^ Positive Selection Kit (Stemcell Technologies) following the manufacturers protocol or PBMCs were cultured directly in RPMI 1640 supplemented with 10% human serum and hMCSF (10–30 ng/mL, R&D Systems) for 7 days at 37 °C, 5% CO_2_ in order to obtain differentiated macrophages as in^59^. Cells were subsequently transfected with siRNA against LXRα β or non-specific scrambled RNA using Dharmafect 4 for 24 hours and activated as indicated. Human cell studies were performed in accordance with approved guidelines by the Ethics Committee of the University College London Hospitals National Health Service Trust. Healthy donors were recruited following their informed consent.

### RNA analysis

BMDM were treated with vehicle, T1317 (1 μ mol/L) or GW3965 (1 μ mol/L) overnight with or without GSK antagonist (1 μ mol/L), followed by LPS activation (*EColi* 0127:B8 or *Salmonella enterica serotype typhimurium*, 100 ng/mL) for 3–6 hours. RNA was extracted and reverse transcription was performed as described^30^ using specific primers (Supplementary Table 1). Gene expression was determined as previously^30^. Each PCR determination was performed in duplicate and each experiment was conducted at least twice as indicated. For human macrophages, cells were activated with LPS (100 ng/ml, *EColi* 026:B6, Sigma) 24 hours after they were fully differentiated.

### Promoter analysis

Luciferase assays were performed as described^30^. Luciferase reporters containing various fragments of the IL-18BP promoter were a kind gift from Dr. Bysani Chandrasekrar (U. Tulane).

### Protein analysis

BMDMs were treated with vehicle or GW3965 (1 μ mol/L) for 24 h in activation medium, followed by Brefeldin A (3.5 μ mol/L) (for IL-18BP) and/or LPS for 6–24 h. Cells were lysed in RIPA buffer with protease inhibitors and protein content was analyzed by Western-blotting. Proteins in supernatants were precipitated using 20% tricholoroacetic acid and incubated overnight at − 20 °C. Antibodies against IL-18BPc (R&D systems, AF129), Hsp90 (Santa Cruz, sc-59577), IL-18 (Biovision, 5180), NLRP3 (Adipogen, MAB7578), Caspase 1 (Millipore 06–503, or Santa Cruz sc-514) and Tubulin (Sigma, T6074) were used. For ELISAs and immunoblotting of supernatants, BMDMs were cultured with vehicle or GW3965 for 6 h and medium was replaced with OptiMEM with LPS (500 ng/mL) and vehicle or GW3965 (1 μ mol/L) for a further 24 hours. ATP was added to a final concentration of 5 mmol/L for the last 2 h. IL-18 and IL-1β levels were analyzed by ELISA (MBL/eBioscience and R&D systems respectively).

### IRF8 Phosphorylation

RAW LXRα WT cells were treated with GW3965 (1 μ mol/L) for 24 hours. Cells were lysed in phospho-lysis buffer as in^56^. Lysates were incubated with antibodies against IRF8 (Santa Cruz, sc-13043x) overnight at 4 °C. Protein complexes containing IRF8 were immunoprecipitated and analysed as in^56^. Antibodies against phospho-Tyrosine coupled to HRP (Merck-Millipore, 4G10), IRF8 (Santa Cruz, sc-6058x) or Hsp90 were used.

### Chromatin Immunoprecipitation

ChIP experiments were performed and analysed as described^30^,^56^ except cells were cross-linked with 1.5 mmol/L of ethylene glycol-bis (succinimidylsuccinate) for 20 min, followed by 10 min with 1% formaldehyde solution. The antibodies employed include: LXR^60^, IRF8 (sc-6058x) and rabbit IgG (Cell signalling, 2729S). The IL-18BP, SREBP1c or β -actin promoters were amplified with primers shown in Supplementary Table 1.

### Statistical analysis

Data is presented as mean ±  SEM when multiple independent experiments (carried out in independent BMDM preparations from different mice) are considered. Data from a representative experiment (of 2 or 3) using an individual BMDM preparation is presented as mean ±  SD of triplicate measurements. Differences were considered significant at *p* <  0.05 using a two-tailed Student t-test.

## Additional Information

**How to cite this article**: Pourcet, B. *et al.* The nuclear receptor LXR modulates interleukin-18 levels in macrophages through multiple mechanisms. *Sci. Rep.* **6**, 25481; doi: 10.1038/srep25481 (2016).

## Supplementary Material

Supplementary Information
