# Supplementary material for: The nuclear receptor LXR modulates interleukin-18 levels in macrophages through multiple mechanisms
Source: Sci Rep. 2016 May 6;6:25481. doi: 10.1038/srep25481 (PMC4858669; doi:10.1038/srep25481)
Supplement: Supplementary Information [file srep25481-s1.pdf]

Supplementary Information

The nuclear receptor LXR modulates interleukin-18 levels in macrophages through multiple mechanisms

Benoit Pourcet[1*#], Matthew C. Gage[1*], Theresa E. León[1,] Kirsty Waddington[1], Oscar M. Pello[1], Knut R Steffensen[2], Antonio Castrillo[3], Annabel F. Valledor[4] and Inés Pineda-Torra[1]

[1] Centre for Clinical Pharmacology, Division of Medicine, University College of London, 5 University Street, London, WC1 E6JF, United Kingdom.

These authors contributed equally to this manuscript

[2] Division of Clinical Chemistry, Department of Laboratory Medicine, Karolinska Institut, Huddinge, Sweden

[3] Instituto de Investigaciones Biomedicas “Alberto Sols” Consejo Superior de Investigaciones Científicas (CSIC) de Madrid, Unidad de Biomedicina (Unidad Asociada al CSIC), Instituto Universitario de Investigaciones Biomedicas y Sanitarias (IUIBS) de la Universidad de Las Palmas de Gran Canaria, Las Palmas, Spain.

[4] School of Biology, University of Barcelona, Diagonal 643, Planta 3, 08028 Barcelona, Spain.

Correspondence should be addressed to:

Inés Pineda-Torra, Centre for Clinical Pharmacology, Division of Medicine, University College London, London WC1E6JF, UK. Phone: +44 (0)20 7679 6535. Fax: +44 (0)20 7679 6211. E-mail: i.torra@ucl.ac.uk

Supplementary Figure S1

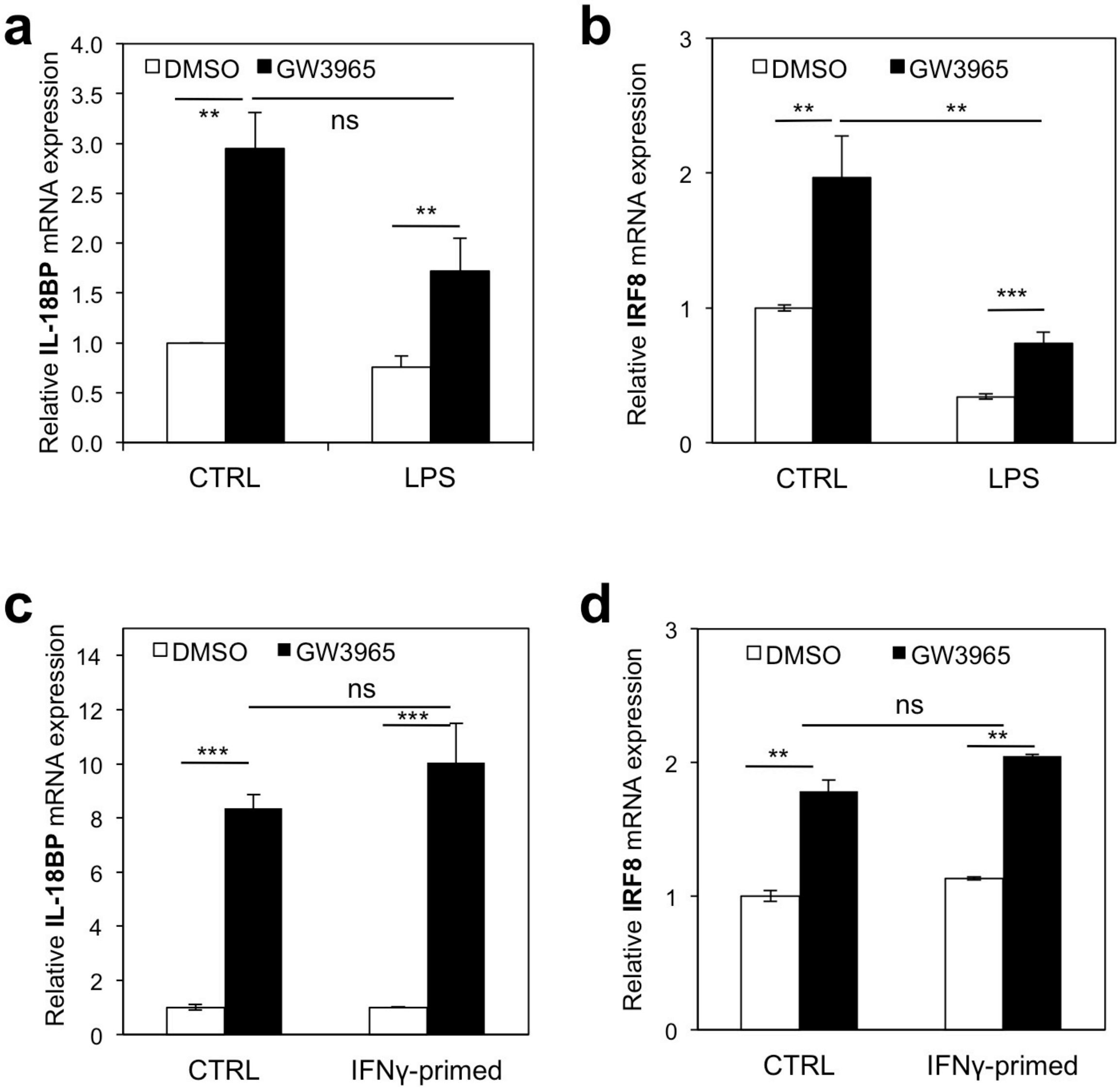

Figure S1: LXR activation decreases IL-18 expression in IFNγ-activated macrophages. **A,B.** BMDM were treated with vehicle (DMSO) or GW3965 (1 μmol/L) for 24h and activated with LPS (100 ng/ml) for the last 6 hours. IL-18BP (A) and IRF8 (B) mRNA levels were analyzed by RT-qPCR. Values indicate expression normalized to cyclophilin and are presented relative to the expression in vehicle-treated cells. Data are mean value ± SD (n=4) (A) or (n=3) (B). **C,D.** BMDM were primed with IFNγ (150 ng/mL) for 3 days, then cells were treated with vehicle (DMSO) or GW3965 (1 μmol/L) for 24 h. IL-18BP (C) and IRF8 (D) mRNA level were analyzed by RT-qPCR as in panel A. Data are mean value ± SD (n=3). Representative experiments are shown. *t*-test: $^{**}p \leq 0.01$, $^{***}p \leq 0.001$

Supplementary Figure S2

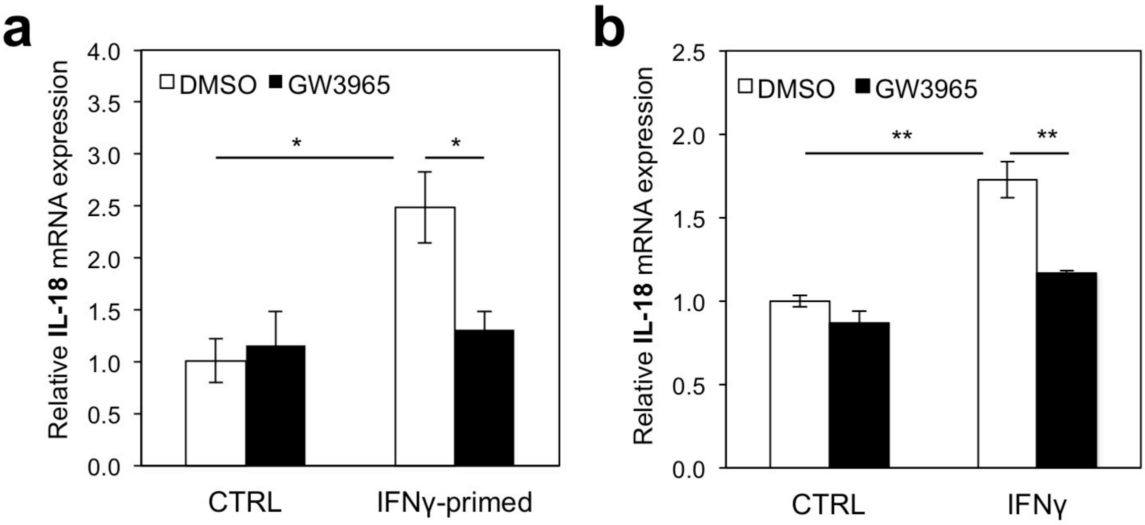

Figure S2: LXR-mediated regulation in LPS- or IFNγ-activated macrophages. **A.** BMDM were primed with IFNγ (150 ng/ml) for 3 days before treatment with vehicle (DMSO) or GW3965 (1 μmol/L) for 24 h. **B.** BMDM were treated with vehicle (DMSO) or GW3965 (1 μmol/L) for 24h and activated with IFNγ (10 ng/ml) for the last 6 hours. IL-18 mRNA level were analyzed by RT-qPCR. For A and B, values indicate expression normalized to cyclophilin and are presented relative to the expression in vehicle-treated cells. Data are mean value ± SD (n=3). Representative experiments are shown. *t*-test: *$p \leq 0.05$, **$p \leq 0.01$.

Supplementary Figure S3

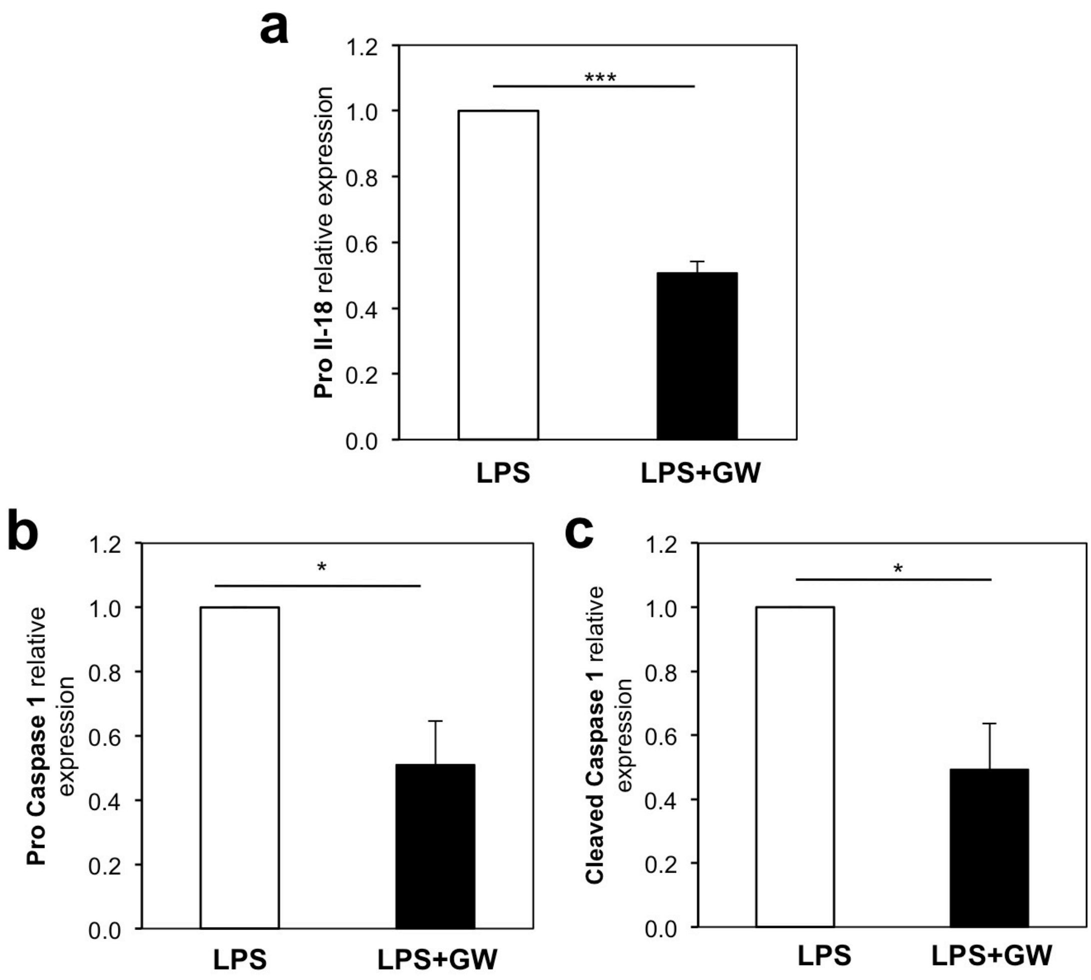

Figure S3: Quantification of the intracellular expression of IL-18 and caspase 1. BMDM were treated with or without GW3965 for 24 hours and activated with LPS for final 6 hours and ATP for the last 2 hours. **A, B, C**, The expression of the indicated proteins were analyzed by immunoblotting and Hsp90 levels were assayed as loading control. Scanned films were quantified by Image J and values indicate expression normalized to Hsp90 and are presented relative to expression in LPS only treated cells, which was set as 1. Data are mean ± SE (n≥3 independent experiments). *t*-test: *$p \leq 0.05$, ***$p \leq 0.001$

New Supplementary Figure S4

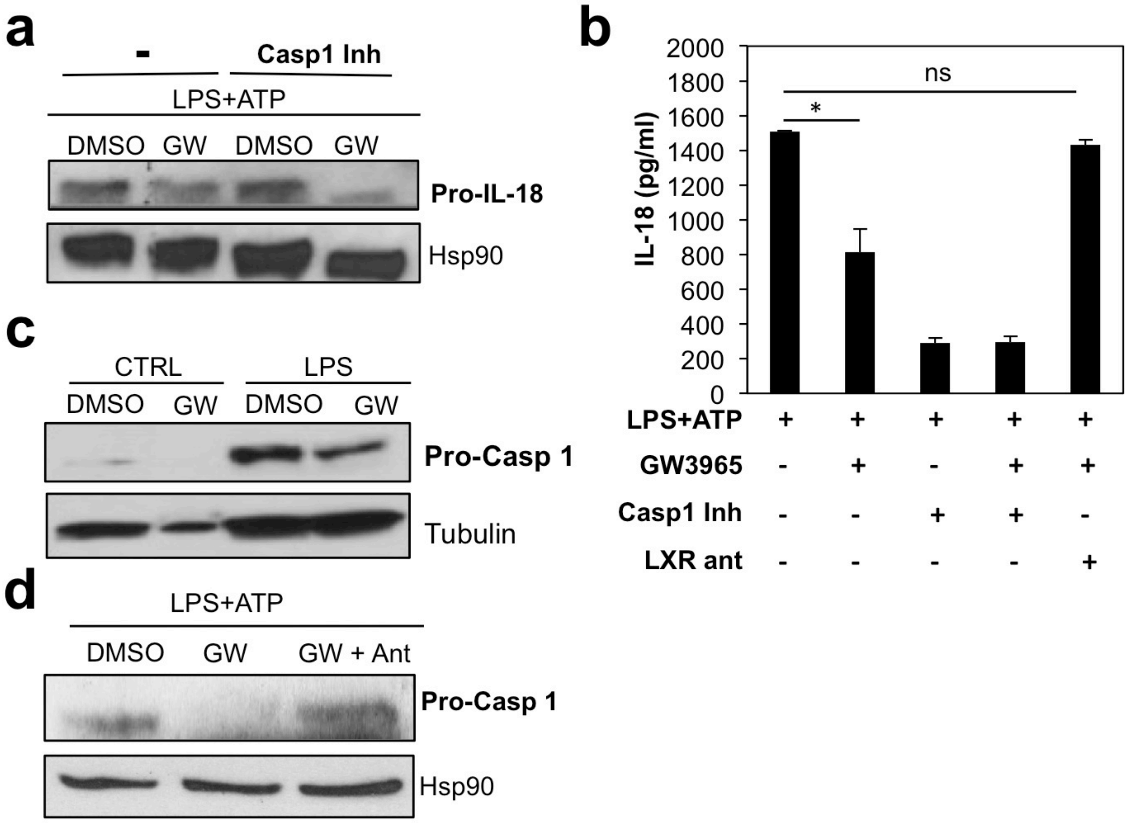

Figure S4: LXR activation modulates expression and maturation of IL-18 levels. Protein expression was analysed by immunoblotting using hsp90 or tubulin as loading controls. **A.** BMDM were pre-treated with or without GW3965 (GW) in the presence of 100 µmol/L Caspase 1 inhibitor Z-WEHD-FMK (Casp Inh) and then activated with LPS with or without ATP. **B.** BMDM were treated as indicated with or without 100 µmol/L Caspase 1 inhibitor (Casp Inh) or the LXR antagonist GSK1440233 (LXR Ant) . Secreted IL-18 levels in culture supernatants were quantified by ELISA. Values represent the mean of 2 independent cell preparations performed in duplicate (mean ± SD) *t*-test: **p*≤0.05, ns *p*>0.05. **C & D.** Intracellular pro-Caspase 1 (pro-Casp1) protein content in LPS- or LPS +ATP-activated macrophages. Were indicated, cells were incubated with LXR antagonist (Ant, 1µM).

Supplementary Figure S5

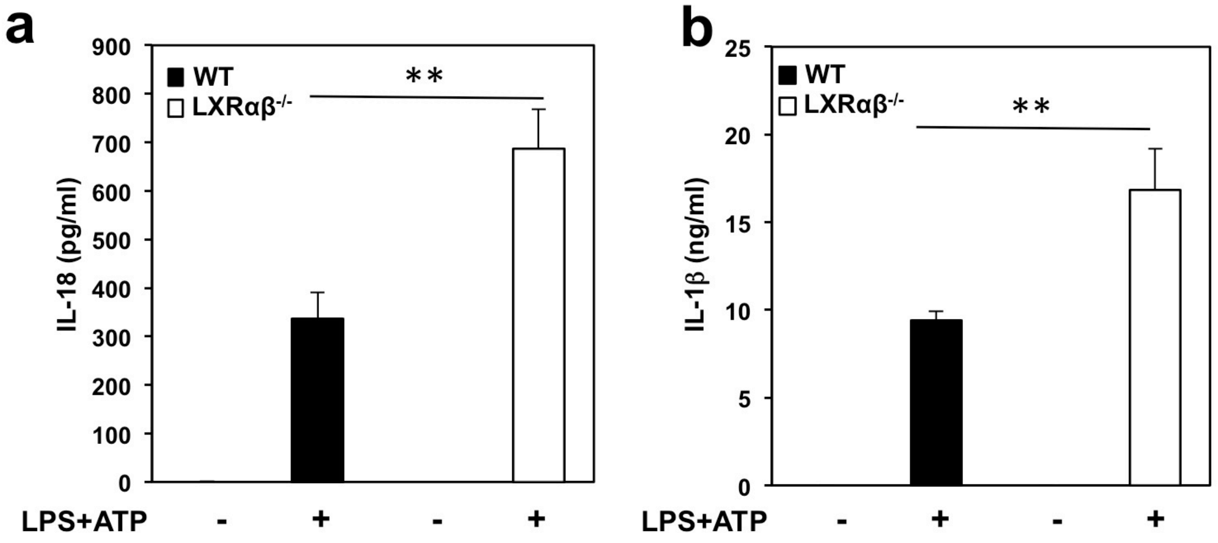

Figure S5: LXR deficiency results in enhanced LPS-activated production of IL-1β and IL-18. BMDM were activated with LPS (100 ng/ml) and ATP for the last 2 hours of culture. Values are the mean ± SD of a representative experiment of two. *t*-test: $^{**}p \leq 0.01$

Supplementary Figure S6

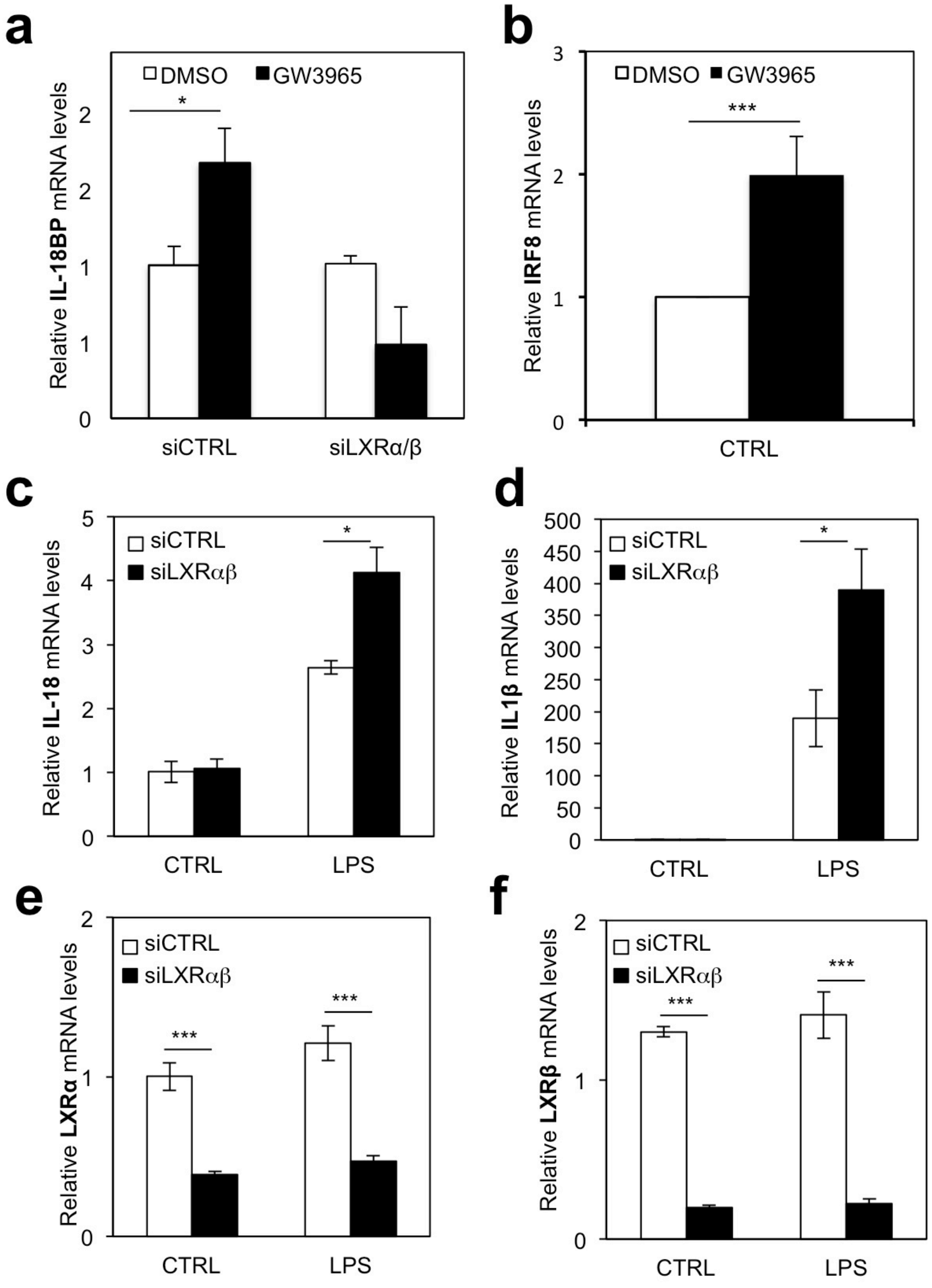

Figure S6: LXR modulates IL18BP, IRF8, IL-18 and IL-1β expression in human primary macrophages. mRNA levels were measured by RT-qPCR, normalised to cyclophilin. **A.** Human macrophages were transfected with siRNA targeting LXRαβ (siLXRαβ) or non-specific scrambled RNA (siCTRL) for 24 hrs before being treated with GW3965 (1 µM) or DMSO as control. Values (mean ± SD, n=3) are presented relative to the expression in corresponding vehicle-treated controls. **B,** Human macrophages were treated with GW3965 for 24 hrs (1 µM) or DMSO as control. Values (mean ± SD, n=5) are presented relative to the expression in vehicle-treated cells which was set as 1. **C, D, E, F.** Human macrophages were transfected with siLXRαβ or siCTRL for 24 hrs before being primed with LPS (100 ng/mL) for 3 hrs. Data are mean value ± SD (n=3). Representative experiments are shown. *t*-test: *p≤0.05, ***p≤0.001.

Supplementary Table 1

Gene	Sequence forward (5'-3')	Sequence reverse (5'-3')
**Mouse RT-qPCR**		
Cyclophilin A	GGCCGATGACGAGCCC	TGTCTTTGGAACTTTGTCTGCAA
CASP1	ACCCTCAAGTTTTGCCCTTT	GATCCTCCAGCAGCAACTTC
IL-1β	TGGGCCTCAAAGGAAAGAAT	CAGGCTTGTGCTGCTTGT
IL-18BP	GCTTAGGAGCCAGAAGCTGA	CTTCCTCTGGGAAGCAACAG
IL-18	ACTTTGGCCGACTTCACTGT	GGGTTCACTGGCACTTTGAT
NLRP3	CCCTTGGAGACACAGGACTC	GAGGCTGCAGTTGTCTAATTCC
**ChIP-qPCR**		
β-actin	ACTATTGGCAACGAGCGGTTC	AAGGAAGGCTGGAAAAGAGCC
Cystatin C promoter	GCAATGACCAACTTCTCTGGTG	CTTACCAGTTCCTCTTCTGTGC
IL-18BP promoter -4.7kb	CTTCGCACTGTCCTCCTACC	CTGAATCCAGTCCTGCTGGT
IL-18BP promoter -1.1kb	GGCTCTGAAAGGGGACTTCT	TTTGGTTCTTGTTGCCTTCA
IL-18BP promoter -0.5kb	GGTGAAGGCTACGTCTATGTTTT	CAGGATCTCTAGGAGTTAGGTCCA
IL-18BP promoter TSS	GCTTGGCTGGGAGTTTTGTA	CCTAAGCCCCGAATAGCTTC
SREBP1c promoter	AGGCTCTTTTCGGGGATGG	TGGGGTTACTGGCGGTCAC
**Human RT-qPCR**		
Cyclophilin A	GCATACGGGTCCTGGCATCTTGTCC	ATGGTGATCTTCTTGCTGGTCTTGC
IL-1β	GACCTGAGCACCTTCTTTCCCTTC	GCAGTTCAGTGATCGTACAGGTGC
IL-18BP	GCTCTGGGCTGGGCTG	AGGGACTGTTCACTCCAGGT
IL-18	GGAAATCGGCCTCTATTTGAAGA	GTCCGGGGTGCATTATCTCT
IRF8	ATTTTTAAGGCCTGGG	AAAGCACAGCGTAACCTCGT
